# Sex-based difference in functional performance and quality of life 1 year after supervised exercise training in patients with symptomatic peripheral artery disease

**DOI:** 10.1177/1358863X251322394

**Published:** 2025-04-30

**Authors:** Stefano Lanzi, Anina Pousaz, Luca Calanca, Lucia Mazzolai

**Affiliations:** Angiology Department, Lausanne University Hospital, University of Lausanne, Lausanne, Switzerland

**Keywords:** claudication, exercise therapy, quality of life, vascular rehabilitation, peripheral artery disease (PAD)

## Abstract

**Introduction:**

The long-term effects of supervised exercise training (SET) on functional performance and health-related quality of life (HRQoL) in symptomatic peripheral artery disease (PAD) are poorly investigated, especially in women. This study investigated these outcomes 1 year after SET in both women and men.

**Methods:**

In this single-arm, prospective, nonrandomized study, patients with symptomatic PAD participating in the 3-month SET program were investigated. Functional performance (6-minute walking distance [6MWD], the stair-climbing test [SCT], and the short physical performance battery [SPPB]), and HRQoL (physical component summary [PCS] score of the Short Form-36 questionnaire) were assessed before and following SET, as well as at 6 and 12 months after SET completion.

**Results:**

Ninety patients (women: *n* = 30; men: *n* = 60) with chronic symptomatic PAD (ankle–brachial index 0.78 ± 0.22; mean age 65.4 ± 10.2 years) were included in the study. The 6MWD (*women*: before: 387.2 ± 88.6 m; after: 472.4 ± 57.0 m; 12 months: 469.9 ± 57.8 m; *men*: before: 431.7 ± 94.0 m; after: 477.5 ± 88.6 m; 12 months: 467.2 ± 73.4 m), SPPB score (*women*: before: 9.6 ± 2.4; after: 11.3 ± 1.0; 12 months: 11.2 ± 0.5; *men*: before: 10.6 ± 1.4; after: 11.5 ± 0.9; 12 months: 11.3 ± 0.8), and SCT (*women*: before: 8.6 ± 4.4 s; after: 5.6 ± 1.6 s; 12 months: 5.8 ± 1.2 s; *men*: before: 6.2 ± 2.3 s; after: 5.0 ± 1.9 s; 12 months: 5.3 ± 1.6 s) significantly improved over time (*p* ≤ 0.001), with no significant differences between women and men. The PCS score (*women*: before: 30.3 ± 8.0; after: 38.8 ± 8.4; 12 months: 35.7 ± 7.4; *men*: before: 32.4 ± 10.5; after: 35.7 ± 9.5; 12 months: 35.4 ± 7.6) significantly improved in women only (*p* = 0.020).

**Conclusion:**

One year after SET, both women and men with PAD exhibit similar functional benefits, whereas HRQoL improvements were observed exclusively in women.

## Introduction

Supervised exercise training (SET) is recommended by guidelines as a first-line therapeutic option for the management of symptomatic patients with chronic peripheral artery disease (PAD).^1–3^ SET is well documented to decrease symptoms and improve walking capacity, functional performance, and quality of life.^1–5^ Most PAD trials reported outcomes after a short-term training intervention period of 3–6 months.^5^ However, the durability of these improvements over time has been less well studied.

Available evidence has demonstrated that treadmill performance and health-related quality of life (HRQoL) are maintained over time following SET programs.^6–10^ On the other hand, functional performance in the months/years following participation in a training intervention has been shown to deteriorate and/or return to baseline values.^11–13^ In the context of PAD, functional performance is usually assessed with clinical tests such as the 6-minute walk test (6MWT), the short physical performance battery (SPPB) test,^3^ and the stair-climbing test (SCT).^14^ It is of paramount importance to maintain high levels of functional performance over time. Indeed, it has previously been shown that poorer baseline functional performance predicts increased mobility loss in patients with PAD.^15^

Sex-based differences in the context of PAD and exercise training have been insufficiently explored, especially in the long-term period. Some authors showed that women with PAD improved treadmill performance less than men following 3^16,17^ or 12^17^ months of SET, whereas others reported equal benefits in both sexes.^18,19^ We recently showed that women and men equally improve functional performance following a 3-month multimodal SET and that women improved HRQoL and self-perceived walking ability to a greater extent than men did.^18^

In this study, we aimed to determine whether functional performance, HRQoL, and self-perceived walking ability were maintained or returned to baseline values 1 year after a 3-month multimodal SET in both women and men.

## Methods

### Patients

Patients with chronic symptomatic atherosclerotic PAD who participated in the 3-month multimodal SET program were included in this single-arm prospective nonrandomized study.^14,20–23^ The inclusion criteria of the SET program were chronic uni- or bilateral lower-limb lifestyle-limiting claudication and a resting ankle–brachial index (ABI) ≤ 0.90 or a posttreadmill drop in the ABI > 20%.^2^ The exclusion criteria were the inability to participate in the SET program three times per week, chronic limb-threatening ischemia, and a cardiac contraindication to exercise. Compared with previous observations,^18,20–23^ patients presenting with atherosclerotic PAD and having a functional performance (including 6MWT, SPPB test, SCT, and maximal gait speed), HRQoL, and self-perceived walking ability assessment up to 1 year following SET were included in the analyses (Supplemental Figure S1). The study was approved by the local ethics committee and was conducted in accordance with the Declaration of Helsinki. All patients provided written informed consent.

### Study design

Patients underwent functional performance (including 6MWT, SPPB test, SCT, and maximal gait speed), HRQoL, self-reported walking ability, and vascular examination at four different times (before SET, after SET, 6 months’ follow up, and 12 months’ follow up; Figure S1).

## Assessments

### Vascular medicine examination

Physical characteristics (body mass index [BMI]), ongoing pharmacological treatment, and cardiovascular risk factors (CVRFs) were assessed. Hemodynamic parameters (resting ABI and toe–brachial index [TBI]) were also recorded.^2^

### Functional performance

The assessment of functional performance was conducted on the same day and time, and always in the following order: (1) 6MWT^24^; (2) SCT^25^; (3) SPPB^26^; and (4) maximal walking speed test.^14^ For a detailed description, please refer to our recent publications.^14,23^

### HRQoL and self-perceived walking ability

Physical and mental HRQoL were evaluated with the generic Medical Outcomes Study Short-Form (SF)-36 questionnaire.^27^ Self-perceived walking abilities were evaluated with the PAD-specific Walking Impairment Questionnaire (WIQ).^28^ The physical (PCS) and mental (MCS) component summaries of the SF-36 were considered for the analyses.^27^ Four subdomains of the WIQ (leg pain, speed, distance, and stair-climbing scores) were considered for the analyses.^28^

### Multimodal SET program

Patients participated in the multimodal SET program combining lower-limb strengthening and outdoor Nordic walking. The multimodal SET program was performed in 30- to 60-minute group sessions (with six to 10 patients) with a training frequency of three times per week and a 3-month duration. Training sessions were supervised by an exercise physiologist and were mainly performed for moderate-to-severe claudication pain (2–3 out of 4 on the claudication pain scale). Additionally, exercise intensity, measured as the rate of perceived exertion (RPE) on Borg’s scale and/or heart rate (HR), was also monitored during the sessions.^3^ Exercise intensity was mainly set between low-to-moderate intensity (RPE: 9–13; %HR_peak_: ≤ 76%). If well tolerated, patients also performed at vigorous intensity (RPE: 14–17; %HR_peak_: 77–95%).^3^ During the multimodal SET program, patients participated in a total of 6 hours of educational workshops on nutrition, physical activity, and CVRFs. The attendance rate was considered satisfactory if the patient completed at least 80% of the sessions within a 3-month period. For an extensive description of the multimodal SET, please refer to our previous publications.^14,18,20–23^

At the end of the multimodal SET program, patients were encouraged to maintain structured walking training, but they were not contacted and were only seen at the planned follow-up visits at 6 and 12 months. During these visits, patients were only asked if they maintained structured walking training at least once a week during the follow-up period.

### Statistical analysis

First, descriptive statistics were performed to report the number of patients who dropped out during the SET program and during the follow-up visits, to describe the baseline characteristics of the patients, and to describe the ongoing pharmacological treatment over the study period. To compare the baseline characteristics of women and men, *t*-tests and chi-squared tests were used.

Subsequently, as previously described,^22,23^ multiple imputations were performed for missing data for patients who failed to complete the multimodal SET program or for those who failed to attend the follow-up visits at 6 and/or 12 months. Multiple imputations were performed to obtain 20 imputed datasets.^22,23^ We used a fully conditional specification with predictive mean matching to impute all variables simultaneously.^29^ Patient characteristics such as age, sex, BMI, CVRFs, comorbidities, ongoing treatment, hemodynamic parameters, and baseline functional performance values were used to impute the datasets.^22,23^

Third, the normality distribution of the residuals was statistically (Kolmogrov–Smirnov test) and visually assessed in the imputed dataset.

Fourth, baseline differences in functional performance, HRQoL, self-perceived walking ability, and hemodynamic parameters between women and men were assessed using *t*-tests. If no baseline differences were found, two-way repeated measures ANOVA (time [before SET, after SET, 6 months, and 12 months] × group [sex]) were performed to assess and compare changes over time between groups. If baseline differences were found, two-way repeated measures ANCOVA were conducted to evaluate the effects of time and group on the outcome variable, while controlling for baseline differences. All models were adjusted for participation in structured walking training during the follow-up period, and for age where appropriate. When two-way ANOVA or ANCOVA revealed a significant time effect or time × group interaction effect, significance was determined using multiple comparisons with Bonferroni adjustment.

The level of significance was set at *p* ≤ 0.05. All the statistical analyses were performed with SPSS 29 software (IBM Corp., Armonk, NY, USA).

## Results

### Participants

Ninety patients (women: *n* = 30; men: *n* = 60) with chronic symptomatic PAD were enrolled in the SET program and were included in the study (Table 1, Figure S1). The baseline characteristics were similar between women and men, except that women were significantly older than men (Table 1). Twenty patients (22%; women: *n* = 8; men: *n* = 12) did not complete the SET program (Figure S1). Seventeen additional patients (women: *n* = 7; men: *n* = 10) did not complete the follow-up visit at 6 months. Finally, six other patients (women: *n* = 2; men: *n* = 4) did not complete the follow-up visit at 12 months. Notably, one patient did not attend the follow-up visit at 6 months for reasons unrelated to the study; the patient did not withdraw and attended the follow-up visit at 12 months.

**Table 1.** Patient characteristics before the multimodal supervised exercise training program.

Variables	Women (*n* = 30)	Men (*n* = 60)	*p*-value
Age, years	69.5 ± 8.1	63.3 ± 10.6	0.006
BMI, kg‧m^–2^	25.9 ± 5.2	27.2 ± 5.3	0.301
Ankle–brachial index	0.73 ± 0.17	0.80 ± 0.23	0.114
Toe–brachial index	0.55 ± 0.18	0.62 ± 0.19	0.076
*Cardiovascular risk factors*			
Hypercholesterolemia	27 (90)	50 (83)	0.396
Hypertension	25 (83)	41 (68)	0.129
Smoking – current	15 (50)	33 (55)	0.328
Smoking – former	10 (33)	23 (38)	0.328
Smoking – never	5 (17)	4 (7)	0.328
Family history of CVD	13 (43)	20 (33)	0.353
Type 1 diabetes	0 (0)	1 (1)	0.477
Type 2 diabetes mellitus	10 (33)	21 (35)	0.875
Prior arterial revascularisation	15 (50)	27 (45)	0.654
*Ongoing medical therapy*			
Antiplatelet	29 (97)	56 (93)	0.515
Antihypertensive	25 (83)	43 (72)	0.225
Lipid lowering	21 (70)	50 (83)	0.144
Anticoagulant	6 (20)	6 (10)	0.188
Antidiabetic	10 (33)	21 (35)	0.875

Data are presented as mean ± SD or *n* (%).

BMI, body mass index; CVD, cardiovascular disease.

Over the study period, pharmacological therapy was similar for each patient, except for: one male patient who stopped antiplatelet therapy; four patients (women: *n* = 1; men: *n* = 3) started anticoagulant therapy; eight patients (women: *n* = 3; men: *n* = 5) who started lipid-lowering therapy; one male patient who stopped receiving antihypertensive therapy; and one male patient who started antihypertensive therapy. During the study period, two patients (women: *n* = 1; men: *n* = 1) stopped smoking and three patients (women: *n* = 1; men: *n* = 2) resumed smoking.

The mean attendance rate of SET was 81% ± 32%. On average, the patients spent 58% ± 19% of the SET program performing Nordic walking and 42% ± 19% of the program performing lower-limb strengthening. Seventy patients (women: *n* = 23; men: *n* = 47) performed the SET program at a low-to-moderate exercise intensity, whereas only 20 patients (women: *n* = 7; men: *n* = 13) were able to achieve vigorous exercise intensity during the second and/or third month of the SET program. There were no adverse events during SET.

Of the 54 patients who attended the follow-up visits at 6 or 12 months, 23 patients (women: *n* = 10; men: *n* = 13) reported taking part in structured walking training at least once a week during the follow-up period.

## Functional performance

### Six-Minute Walk Test (6MWT)

Baseline values significantly differed between groups (*p* = 0.034; Table 2). Two-way ANCOVA revealed a significant time with no significant group and group × time interaction effect for the 6-minute walk distance (6MWD). Multiple comparison analyses revealed that, compared to before SET, the 6MWD significantly improved following SET (*p* ≤ 0.001), at 6 months (*p* ≤ 0.001), and at 12 months (*p* ≤ 0.001) in both women and men. Compared to after SET, there was no significant difference in the 6MWD at 6 months (*p* = 0.299) or at 12 months (*p* > 0.99). There was no significant difference in the 6MWD between 6 and 12 months (*p* > 0.99).

**Table 2.** Functional performance before and after the multimodal supervised exercise training program and at the 6- and 12-month follow ups.

Variable	Group	Before SET	After SET	6 months	12 months	Time effect	Group effect	Group × time effect
6MWD, m	Women	387.2 ± 88.6^a^	472.4 ± 57.0^b^	469.5 ± 60.6^b^	469.9 ± 57.8^b^	⩽ 0.001	0.199	0.520
	Men	431.7 ± 94.0	477.5 ± 88.6^b^	463.9 ± 88.3^b^	467.2 ± 73.4^b^			
Performance on 12-stair flight^c^, s	Women	8.6 ± 4.4^a^	5.6 ± 1.6^b^	5.8 ± 1.0^b^	5.8 ± 1.2^b^	⩽ 0.001	0.670	0.514
	Men	6.2 ± 2.3	5.0 ± 1.9^b^	5.5 ± 2.0^b^	5.3 ± 1.6^b^			
Total SPPB score^c^	Women	9.6 ± 2.4^a^	11.3 ± 1.0^b^	11.3 ± 0.6^b^	11.2 ± 0.5^b^	⩽ 0.001	0.472	0.406
	Men	10.6 ± 1.4	11.5 ± 0.9^b^	11.3 ± 1.0^b^	11.3 ± 0.8^b^			
Maximal gait speed, m‧s^–1^	Women	1.40 ± 0.29^a^	1.64 ± 0.24^b^	1.57 ± 0.18	1.58 ± 0.22	0.020	0.201	0.655
	Men	1.59 ± 0.33	1.76 ± 0.38^b^	1.59 ± 0.42	1.61 ± 0.33			

Data are presented as mean ± SD.

All models were adjusted for participation in structured walking training at least once a week during the follow-up period.

a *p* ⩽ 0.05 for significant baseline difference to men.

b *p* ⩽ 0.05 for significant difference to before SET.

c Adjusted for age.

6MWD, 6-minute walking distance; SET, supervised exercise training; SPPB, short physical performance battery.

### Stair-climbing test (SCT)

Baseline values significantly differed between groups (*p* ≤ 0.001; Table 2). Two-way ANCOVA revealed a significant time effect with no significant group and group × time interaction effect for the SCT performance. Multiple comparison analyses revealed that, compared to that before SET, SCT performance improved following SET (*p* ≤ 0.001), at 6 months (*p* ≤ 0.001), and at 12 months (*p* ≤ 0.001) in both women and men. Compared to after SET, there was no significant difference in SCT performance at 6 months (*p* = 0.304) or at 12 months (*p* > 0.99). There was no significant difference in SCT performance between 6 and 12 months (*p* > 0.99).

### Short physical performance battery (SPPB)

Baseline values significantly differed between groups (*p* = 0.010; Table 2). Two-way ANCOVA revealed a significant time effect with no significant group and group × time interaction effect for the SPPB total score. Multiple comparison analyses revealed that, compared to before SET, the SPPB total score improved following SET (*p* ≤ 0.001), at 6 months (*p* ≤ 0.001), and at 12 months (*p* ≤ 0.001) in both women and men. Compared to after SET, there was no significant difference in SPPB total score at 6 months (*p* = 0.455) or at 12 months (*p* > 0.99). There was no significant difference in SPPB total score between 6 and 12 months (*p* > 0.99).

### Maximal walking speed test

Baseline values significantly differed between groups (*p* = 0.008; Table 2). Two-way ANCOVA revealed a significant time effect with no significant group and group × time interaction effect for the maximal walking speed. Multiple comparison analyses revealed that, compared to before SET, the maximal walking speed improved in both women and men following SET (*p* = 0.004) with no difference at 6 months (*p* = 0.598) and at 12 months (*p* = 0.059). Compared to after SET, there was no significant difference in the maximal walking speed at 6 months (*p* = 0.596) or at 12 months (*p* > 0.99). There was no significant difference in the maximal walking speed between 6 and 12 months (*p* > 0.99).

### Health-related quality of life (HRQoL)

Baseline PCS score values were similar between groups (*p* = 0.338; Supplemental Table S1). Two-way ANOVA revealed a significant time effect with no significant group effect and a significant group × time interaction effect for the PCS score. Multiple comparison analyses revealed that, compared to before SET, the PCS score significantly improved following SET (*p* = 0.011), and at 6 months (*p* = 0.001) and 12 months (*p* = 0.045) in women only. There was no significant difference in PCS score over time among men.

Baseline MCS score values were similar between groups (*p* = 0.636; Table S1). There was no significant difference in MCS score over time in both women and men.

### Self-perceived walking ability

Baseline WIQ leg pain, WIQ speed, WIQ distance, and WIQ stair-climbing scores were similar between groups (*p* ≥ 0.157; Table S1).

Two-way ANOVA revealed a significant time effect with no significant group or group × time interaction effect for the WIQ leg pain score. Multiple comparison analyses revealed that the WIQ leg pain score significantly improved at 6 months in both women and men (*p* = 0.049) with no significant changes following SET (*p* = 0.138) and at 12 months (*p* = 0.309). Compared to those after SET, there were no significant improvements in the WIQ leg pain score at 6 months (*p* > 0.99) or at 12 months (*p* > 0.99). Compared to those at 6 months, there were no significant improvements in the WIQ leg pain score at 12 months (*p* > 0.99).

Two-way ANOVA revealed a significant time effect with no significant group or group × time interaction effect for the WIQ speed score. Multiple comparison analyses revealed that the WIQ speed score significantly improved following SET (*p* = 0.050) and at 6 months (*p* = 0.050) in both women and men with no significant changes at 12 months (*p* = 0.132). Compared to those after SET, there were no significant improvements in the WIQ speed score at 6 months (*p* > 0.99) or at 12 months (*p* > 0.99). Compared to those at 6 months, there were no significant improvements in the WIQ speed score at 12 months (*p* > 0.99).

Two-way ANOVA revealed a significant time effect with no group effect and with a significant group × time interaction effect for the WIQ distance score. Multiple comparison analyses revealed that, compared to before SET, the WIQ distance score significantly improved following SET (*p* = 0.050), at 6 months (*p* ≤ 0.001), and 12 months (*p* = 0.011) in women only. There was no significant difference in the WIQ distance score over time among men.

Two-way ANOVA revealed a significant time effect with no group effect and with a significant group × time interaction effect for the WIQ stair-climbing score. Multiple comparison analyses revealed that, compared to before SET, the WIQ stair-climbing score significantly improved following SET (*p* = 0.005), at 6 months (*p* = 0.028), and 12 months (*p* = 0.037) in women only. There was no significant difference in the WIQ stair-climbing score over time among men.

### Hemodynamic parameters

Baseline ABI, TBI, and posttreadmill ABI drop in the most symptomatic leg were similar between groups (*p* ≥ 0.076; Table S2). Two-way ANOVA revealed no significant time, group, and group × time interaction effect for the ABI in the most symptomatic leg. Two-way ANOVA revealed no significant time and group × time interaction effect, with a significant group effect for the TBI in the most symptomatic leg. The two-ways ANOVA showed a significant time effect with no significant group and group × time interaction effect for the posttreadmill ABI drop in the most symptomatic leg. Multiple comparison analyses revealed that the posttreadmill ABI drop in the most symptomatic leg significantly decreased at 12 months when compared to before SET (*p* = 0.013).

### Physical characteristics

Baseline BMI was similar between groups (*p* = 0.301). Two-way ANOVA revealed no significant time (*p* = 0.426), group (*p* = 0.467), and group × time interaction (*p* = 0.455) effect for the BMI (before SET, women: 25.9 ± 5.2 kg‧m^–2^, men: 27.2 ± 5.3 kg‧m^–2^; after SET, women: 27.0 ± 5.5 kg‧m^–2^, men: 27.5 ± 4.9 kg‧m^–2^; at 6 months, women: 27.8 ± 4.7 kg‧m^–2^, men: 27.1 ± 4.7 kg‧m^–2^; at 12 months, women: 27.6 ± 4.5 kg‧m^–2^, men: 27.0 ± 4.4 kg‧m^–2^).

## Discussion

The main findings of the present investigation are that the improvements in functional performance previously observed following the 3-month multimodal SET program were similarly maintained over a 12-month period in both women and men. Additionally, PCS score and self-perceived walking ability were found to be improved over a 12-month period in women only.

Compared to men, women have a greater prevalence of atypical lower-limb symptoms, potentially contributing to low PAD detection and delayed therapeutic care initiation.^30^ Additionally, women have been found to have lower functional performance than men.^31–33^ Consistent with these findings, in the present investigation, women were older, had more severe PAD (lower ABI and TBI, although not significant), and presented with lower baseline functional performance than men. Despite this, we previously showed that women and men equally improve functional performance following a 3-month multimodal SET and that women improved their HRQoL and self-perceived walking ability to a greater extent than men did.^18^ The novelty of the present investigation is that the improvements in functional performance were similarly maintained over a 12-month period in both women and men, except for the maximal walking speed, which decreased during the follow-up period and was no longer significant. Additionally, PCS score and two subdomains of the WIQ (distance and stair-climbing scores) were significantly improved only in women over the study period. Our results are in line with previous investigations reporting equal benefits in functional performance (only assessed by 6MWT) in both sexes following a functional, pain-free, home-based exercise training program.^19^ On the other hand, our results contrast with previous findings showing that functional performance deteriorates or returns to baseline values in the follow-up period following a supervised treadmill training.^12,13^ Our results also contrast with those of Gommans et al.,^17^ who reported that HRQoL was unchanged in both women and men and that self-perceived walking ability was significantly lower in women following SET (primarily based on treadmill training). It is interesting to note that, although not significant, mental HRQoL slightly improved following the multimodal SET program but decreased in the follow-up period in both women and men. A possible explanation is that the social/mental support of group-based exercise sessions during the multimodal SET program may contribute to improved mental HRQoL but that this positive effect fades when patients stop participating in group sessions during the follow-up period. The reasons for the heterogeneity among studies are unknown, but it may be related to the patients’ baseline characteristics and/or the training program/modalities. Indeed, in the present investigation, patients participated in a multimodal functional training program. It is therefore possible that these training modalities are responsible for the persistence of the improvements over time. This highlights the need for future research.

It has recently been shown that participating actively in a home-based exercise training program is associated with a lower rate of death at the 7-year follow up in patients with PAD, especially among women.^34^ These findings highlight the pivotal role of vascular rehabilitation in women with PAD and suggest that women should be highly encouraged to participate in training interventions.^3^ Interestingly, a recent observation reported that when enrolled in SET, women reported higher rates of participation than men did.^35^

Despite most data demonstrating the ABI did not significantly change following exercise interventions,^5,36,37^ a meta-analysis also showed that ABI was significantly improved (mean difference 0.06) following a 3-month exercise therapy.^36^ Accordingly, in the present investigation, we observed a progressive increase in ABI values over the study period (although not significant), especially in women (Table S2).

### Study limitations

This study is not without limitations. First, the study did not include a control group that did not participate in the 3-month multimodal SET program. Second, the outcomes were collected over a relatively short time, and further research is needed for long-term (> 5 years) training adaptations. Third, during the follow-up period, patients were encouraged to maintain structured walking training, but the total amount of physical activity and training characteristics (volume, intensity, type) was not monitored. Indeed, patients who continue to exercise in the follow-up period have been shown to maintain benefits and report improved HRQoL.^38^ Similarly, patients with PAD who first participated in a 3-month SET program and then translated to a home-based exercise training program during the follow-up period have been shown to continue improving their walking performance over time.^39^ In the present investigation, 23 patients reported taking part in structured walking training at least once a week during the follow-up period. Based on these considerations, all statistical models were adjusted for participation in structured walking training during the follow-up period. Fourth, multiple imputations were performed to improve the statistical power. Interestingly, the results were similar when analyses were performed without multiple imputations (Table S3).

## Conclusion

The results presented herein demonstrate that the improvements in functional performance (6MWT, SPPB, and SCT) previously observed following the 3-month multimodal SET program were similarly maintained over a 12-month period in both women and men. Additionally, HRQoL and self-perceived walking ability were found to be improved over a 12-month period in women only. Women should be highly encouraged to participate in training interventions.

## Supplemental Material

sj-docx-1-vmj-10.1177_1358863X251322394 – Supplemental material for Sex-based difference in functional performance and quality of life 1 year after supervised exercise training in patients with symptomatic peripheral artery disease Supplemental material, sj-docx-1-vmj-10.1177_1358863X251322394 for Sex-based difference in functional performance and quality of life 1 year after supervised exercise training in patients with symptomatic peripheral artery disease by Stefano Lanzi, Anina Pousaz, Luca Calanca and Lucia Mazzolai in Vascular Medicine
