# Supplementary material for: Sex-based difference in functional performance and quality of life 1 year after supervised exercise training in patients with symptomatic peripheral artery disease
Source: Vasc Med. 2025 Apr 30;30(4):423–30. doi: 10.1177/1358863X251322394 (PMC12334798; doi:10.1177/1358863X251322394)
Supplement: sj-docx-1-vmj-10.1177_1358863X251322394 – Supplemental material for Sex-based difference in functional performance and quality of life 1 year after supervised exercise training in patients with symptomatic peripheral artery disease [file sj-docx-1-vmj-10.1177_1358863X251322394.docx]

**Supplemental Figure S1**. Study flow diagram.

**
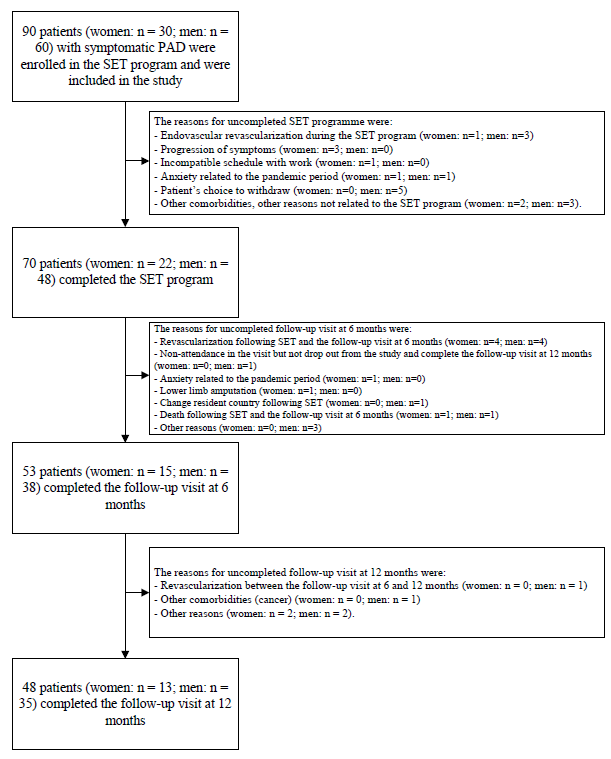
**

PAD = peripheral artery disease; SET = supervised exercise training.

**Supplemental Table S1.** Health-related quality of life and self-perceived walking ability before and after the multimodal SET program and at 6- and 12-month follow-up.

| **Variable** | **Group** | **Before SET** | **After SET** | **6 months** | **12 months** | **Time effect** | **Group effect** | **Group x Time effect** |
| --- | --- | --- | --- | --- | --- | --- | --- | --- |
| *Health-related quality of life* |  |  |  |  |  |  |  |  |
| SF-36 PCS | Women | 30.3 ± 8.0 | 38.8 ± 8.4* | 37.9 ± 7.0* | 35.7 ± 7.4* | 0.004 | 0.335 | 0.020 |
|  | Men | 32.4 ± 10.5 | 35.7 ± 9.5 | 34.7 ± 8.3 | 35.4 ± 7.6 |  |  |  |
| SF-36 MCS | Women | 40.1 ± 10.4 | 44.4 ± 9.6 | 40.9 ± 7.5 | 42.6 ± 6.4 | 0.105 | 0.522 | 0.471 |
|  | Men | 41.3 ± 12.1 | 45.4 ± 9.9 | 41.8 ± 10.2 | 41.8 ± 8.4 |  |  |  |
| *Self-perceived walking ability* |  |  |  |  |  |  |  |  |
| WIQ leg pain score - % | Women | 58.2 ± 17.7 | 66.0 ± 15.6 | 72.2 ± 8.6* | 69.3 ± 10.4 | 0.038 | 0.282 | 0.112 |
|  | Men | 58.0 ± 22.9 | 64.6 ± 15.9 | 64.2 ± 17.2* | 63.8 ± 14.5 |  |  |  |
| WIQ speed score - % | Women | 55.3 ± 22.9 | 67.7 ± 16.6* | 69.3 ± 13.3* | 69.8 ± 15.4 | 0.010 | 0.285 | 0.160 |
|  | Men | 58.3 ± 24.5 | 64.8 ± 20.3* | 62.2 ± 18.4* | 63.9 ± 20.2 |  |  |  |
| WIQ distance score - % | Women | 60.1 ± 21.7 | 72.5 ± 15.5* | 76.2 ± 7.7* | 74.3 ± 13.8* | ≤0.001 | 0.504 | 0.010 |
|  | Men | 64.3 ± 21.6 | 67.9 ± 19.6 | 70.7 ± 15.2 | 68.7 ± 16.5 |  |  |  |
| WIQ stair climbing score - % | Women | 54.6 ± 22.9 | 70.7 ± 17.1* | 71.7 ± 11.0* | 72.3 ± 13.1* | 0.001 | 0.157 | 0.050 |
|  | Men | 61.5 ± 21.0 | 68.2 ± 17.9 | 64.3 ± 19.7 | 66.9 ± 15.2 |  |  |  |

Data are presented as mean ± standard deviation.

All models were adjusted for participation in a structured walking training at least once a week during the follow-up period.

*P⩽0.05 for significant difference to before SET.

SET = supervised exercise training; SF-36 = The 36-Item Short Form Health Survey; PCS = physical component summary score of the SF-36 questionnaire; MCS = mental component summary score of the SF-36 questionnaire; WIQ = walking impairment questionnaire.

**Supplemental Table S2.** Hemodynamic parameters before and after the multimodal SET program and at 6- and 12-month follow-up.

| **Variable** | **Group** | **Before SET** | **After SET** | **6 months** | **12 months** | **Time effect** | **Group effect** | **Group x Time effect** |
| --- | --- | --- | --- | --- | --- | --- | --- | --- |
| ABI most symptomatic leg | Women | 0.73 ± 0.17 | 0.75 ± 0.13 | 0.81 ± 0.15 | 0.83 ± 0.13 | 0.872 | 0.251 | 0.741 |
|  | Men | 0.80 ± 0.23 | 0.82 ± 0.16 | 0.83 ± 0.18 | 0.82 ± 0.18 |  |  |  |
| TBI most symptomatic leg ^a^ | Women | 0.55 ± 0.18 | 0.58 ± 0.14 | 0.61 ± 0.11 | 0.55 ± 0.10 | 0.635 | 0.049 | 0.703 |
|  | Men | 0.62 ± 0.19 | 0.61 ± 0.16 | 0.63 ± 0.15 | 0.59 ± 0.15 |  |  |  |
| Post-treadmill ABI drop most symptomatic leg - % | Women | 46.3 ± 19.9 | 45.6 ± 17.3 | 43.7 ± 14.4 | 36.0 ± 18.6* | 0.004 | 0.931 | 0.159 |
|  | Men | 46.1 ± 24.5 | 45.9 ± 20.3 | 43.4 ± 17.9 | 37.4 ± 19.4* |  |  |  |

Data are presented as mean ± standard deviation.

All models were adjusted for participation in a structured walking training at least once a week during the follow-up period. ^a^ adjusted for age.

*P⩽0.05 for significant difference to before SET.

ABI = ankle-brachial index; TBI = toe brachial index; SET =supervised exercise training

**Supplemental Table S3.** Functional performance, health-related quality of life, and self-perceived walking ability before and after the multimodal supervised exercise training (SET) program and at 6- and 12-month follow-up (without multiple imputations).

| **Variable** | **Group** | **Before SET** | **After SET** | **6 months** | **12 months** | **Time effect** | **Group effect** | **Group x Time effect** |
| --- | --- | --- | --- | --- | --- | --- | --- | --- |
| 6MWD – m | Women | 387.2 ± 88.6^$^  n=30 | 471.2 ± 66.9*  n=22 | 473.1 ± 90.3*  n=14 | 471.2 ± 89.8*  n=13 | ≤0.001 | 0.100 | 0.100 |
|  | Men | 433.3 ± 98.1  n=55 | 478.0 ± 105.0*  n=43 | 462.9 ± 116.3*  n=35 | 466.6 ± 106.5*  n=29 |  |  |  |
| Performance on 12-stair flight ^a^ - s | Women | 8.6 ± 4.4^$^  n=30 | 5.8 ± 1.8*  n=22 | 6.0 ± 1.4*^#^  n=14 | 6.1 ± 1.7*^#^  n=13 | ≤0.001 | 0.260 | 0.234 |
|  | Men | 6.1 ± 2.4  n=55 | 5.0 ± 2.3*  n=43 | 5.4 ± 2.7*^#^  n=34 | 5.1 ± 2.3*^#^  n=29 |  |  |  |
| Total SPPB score ^a^ | Women | 9.6 ± 2.4^$^  n=30 | 11.3 ± 1.2*  n=22 | 11.5 ± 0.9*  n=14 | 11.2 ± 0.7*  n=13 | ≤0.001 | 0.355 | 0.226 |
|  | Men | 10.7 ± 1.5  n=55 | 11.6 ± 1.1*  n=44 | 11.3 ± 1.3*  n=33 | 11.3 ± 1.2*  n=29 |  |  |  |
| Maximal gait speed – m^.^s^-1^ | Women | 1.37 ± 0.29^$^  n=28 | 1.59 ± 0.26*  n=22 | 1.49 ± 0.20  n=14 | 1.55 ± 0.32*  n=13 | ≤0.001 | 0.102 | 0.867 |
|  | Men | 1.59 ± 0.34  n=55 | 1.77 ± 0.44*  n=44 | 1.63 ± 0.53  n=35 | 1.65 ± 0.46*  n=29 |  |  |  |
| SF-36 PCS | Women | 30.2 ± 8.4  n=27 | 39.3 ± 9.5*  n=23 | 38.5 ± 12.9*  n=14 | 35.9 ± 11.5  n=13 | 0.009 | 0.188 | 0.046 |
|  | Men | 32.6 ± 11.2  n=52 | 35.4 ± 11.0  n=45 | 33.9 ± 10.8  n=35 | 34.6 ± 11.3  n=33 |  |  |  |
| SF-36 MCS | Women | 40.0 ± 11.0  n=27 | 44.2 ± 11.0  n=23 | 38.0 ± 13.7^#^  n=14 | 43.2 ± 9.8  n=13 | 0.011 | 0.716 | 0.252 |
|  | Men | 41.3 ± 13.0  n=52 | 45.6 ± 11.5  n=45 | 42.0 ± 13.4^#^  n=35 | 40.7 ± 12.6  n=33 |  |  |  |
| WIQ leg pain score - % | Women | 58.3 ± 18.7  n=27 | 66.3 ± 17.9  n=23 | 77.7 ± 10.0*  n=14 | 73.1 ± 15.2  n=13 | 0.025 | 0.151 | 0.065 |
|  | Men | 57.9 ± 24.6  n=52 | 64.4 ± 18.5  n=45 | 62.5 ± 22.5*  n=35 | 62.5 ± 19.8  n=32 |  |  |  |
| WIQ speed score - % | Women | 55.2 ± 24.1  n=27 | 68.3 ± 19.0*  n=23 | 74.7 ± 18.2  n=14 | 74.2 ± 23.1  n=13 | 0.028 | 0.022 | 0.185 |
|  | Men | 58.5 ± 26.3  n=52 | 64.5 ± 23.5*  n=45 | 69.2 ± 20.1  n=35 | 62.3 ± 27.7  n=32 |  |  |  |
| WIQ distance score - % | Women | 59.9 ± 22.9  n=27 | 73.4 ± 17.7*  n=23 | 80.1 ± 10.0*  n=14 | 79.7 ± 20.1*  n=13 | ≤0.001 | 0.108 | 0.008 |
|  | Men | 64.6 ± 23.2  n=52 | 67.4 ± 22.7  n=45 | 60.3 ± 24.4  n=34 | 67.0 ± 22.6  n=32 |  |  |  |
| WIQ stair climbing score - % | Women | 53.7 ± 25.0  n=25 | 71.4 ± 20.5*  n=21 | 76.8 ± 14.7*  n=14 | 76.9 ± 19.3*  n=13 | 0.001 | 0.980 | 0.020 |
|  | Men | 61.9 ± 22.7  n=51 | 67.8 ± 21.2  n=43 | 62.2 ± 26.6  n=33 | 65.3 ± 21.1  n=31 |  |  |  |

Data are presented as mean ± standard deviation.

Baseline differences between women and men were assessed using T-tests. If no baseline differences were found, linear mixed models (group (women vs men) × time (before SET vs after SET vs 6 month and 12 months follow-up) were performed to assess and compare changes over time between groups. If baseline differences were found, linear mixed models were conducted to evaluate the effects of time and group on the outcome variable, while controlling for baseline differences. All models were adjusted for participation in a structured walking training during the follow-up period, and for age where appropriate. Significance was determined using multiple comparisons with Bonferroni adjustment.

^$^P ⩽ 0.05 for significant baseline difference to men; *P⩽0.05 for significant difference to before SET; ^#^P ⩽ 0.05 for significant difference to after SET. ^a^ adjusted for age.

SET = supervised exercise training; 6MWD = six-minute walking distance; SPPB = Short Physical Performance Battery; SF-36 = The 36-Item Short Form Health Survey; PCS = physical component summary score of the SF-36 questionnaire; MCS = mental component summary score of the SF-36 questionnaire; WIQ = walking impairment questionnaire.
